# Rupture Behavior of Paper Sheets Immersed in Carboxymethyl Cellulose Aqueous Solutions

**DOI:** 10.3390/polym18172052

**Published:** 2026-08-24

**Authors:** Mohamed Hussien, Rentaro Kanamori, Jie Liu, Joon Yang Kim, Tatsuo Kaneko, Mika Kawai, Tetsu Mitsumata

**Affiliations:** 1 Graduate School of Science and Technology, Niigata University, Niigata 950-2181, Japan; 2 Key Laboratory of Synthetic and Biological Colloids, Ministry of Education, School of Chemical and Material Engineering, Jiangnan University, Wuxi 214122, China; 3 Graduate School of Modern Society and Culture, Niigata University, Niigata 950-2181, Japan

**Keywords:** paper sheet, cellulose, mechanical properties

## Abstract

The mechanical characteristics and the rupture behavior of dry paper and papers immersed in pure water or a carboxymethyl cellulose (CMC) aqueous solution were investigated by measuring the shape changes due to the immersion and by uniaxial tensile tests. The weight changes and dimensional changes for these samples due to the immersion were evaluated by the gravimetric method and image analysis, respectively. The absorption ratio of the paper was 2.3 for pure water, and it increased up to 2.7 with the CMC concentration. A deformation of 5% at maximum was observed in the direction perpendicular to the fiber orientation due to the absorption. All the samples demonstrated similar stress–strain curves in the regions of linear viscoelasticity and plastic deformation. The peak stress, Young’s modulus, and strain energy density of CMC wet paper showed lower values than those of water wet paper, while the peak strain was the same for both samples. Similar behavior was found in the cross direction, although the difference was not significant. These results strongly indicate that the penetration and adsorption of CMC molecules lead to a large expansion, resulting in the disentanglement of paper fibers and a significant reduction in the mechanical properties due to the strong fluid lubrication effect.

## 1. Introduction

Fragile cultural artifacts, such as animation cels and ancient papyrus, hold massive historical and artistic value. Despite differences in their forms, materials, and origins, both represent irreplaceable records of human creativity and knowledge. Animation cels—widely used in the production of animated films from the 1950s to the early 2000s—are composed of cellulose acetate sheets hand-painted with acrylic paints [1,2,3,4]. Over the decades, they have gained significance not only as production materials but also as collectible artworks and historical documents. Similarly, ancient papyrus manuscripts preserve the writings and illustrations of early civilizations, offering essential insight into the development of human history, knowledge, and culture [5]. Although these two materials differ in their era and composition, they have traditionally been preserved using similar techniques, most notably the application of a backing paper to provide structural support and stability [6,7]. However, over time, the backing paper itself undergoes degradation [8], which can compromise the integrity of the primary object. This deterioration often leads to challenges in separating the layers without damage, making the conservation process more complex. In both animation cels and ancient papyrus, the adhesion between the backing paper and the artifact becomes problematic as aging progresses. With an increasing emphasis on digitization and sustainable conservation, there is a growing need to revisit and refine these traditional preservation methods.

We have investigated, so far, the physico-chemical interaction between paper sheets and various polymers. In our previous paper, significant increases in the mechanical properties were observed for a paper sheet composite with CMC [9]; the breaking stress, breaking strain, and breaking energy for a paper sheet composite obtained by immersing a sheet in 3 wt.% CMC aqueous solution were 2.0-, 3.9-, and 8.0-fold higher than those for untreated paper, respectively. On the other hand, Young’s modulus for the CMC wet papers was comparable with that for the untreated paper; only the rupture behavior was changed. Furthermore, the morphological studies indicated that the CMC penetrated into the inner part of the paper and covered the paper fibrils. Therefore, the significant improvement in the breaking strength and breaking strain of CMC wet papers was considered to be caused by the interconnection and elongation of CMC covering the paper fibrils [10]. These findings demonstrated that the strength of paper can be dramatically increased through such a simple treatment. This technique is useful, for example, for repairing the deterioration of backing paper that has been used in the restoration of valuable artifacts. Its application, by simply brushing or spraying a polymer solution onto deteriorated backing paper, can enhance the paper’s strength and further deterioration can be prevented.

On the other hand, the backing paper may sometimes be severely deteriorated. In such cases, it is necessary to completely remove the backing paper from an artifact. However, backing paper breaks when peeled off in a dry state, and its fragments frequently remain on an artifact. It is well known that the interactions between paper fibers are greatly reduced by wetting. Thus, the study of mechanical properties of paper in a wet state is also useful for completely removing paper fragments.

In this paper, we focus on the mechanical properties of paper in a wet state and the effect of CMC addition on these properties. CMC is a polymer material currently used widely in the field of artifact conservation and restoration [11]. CMC generally possesses high flexibility and exhibits a low shrinkage rate during long-term storage. It also has advantages such as resistance to pests and mildew. Uniaxial tensile tests were performed on paper sheets in a wet state in water and on paper sheets immersed in CMC aqueous solution. The mechanical parameters, such as rupture strain, rupture stress, Young’s modulus, and strain energy density, were analyzed. Furthermore, to systematically understand the rupture behavior of paper sheets, similar experiments were conducted on paper sheets in a dry state.

## 2. Experimental Procedures

### 2.1. Sample Preparation

**Figure 1:**
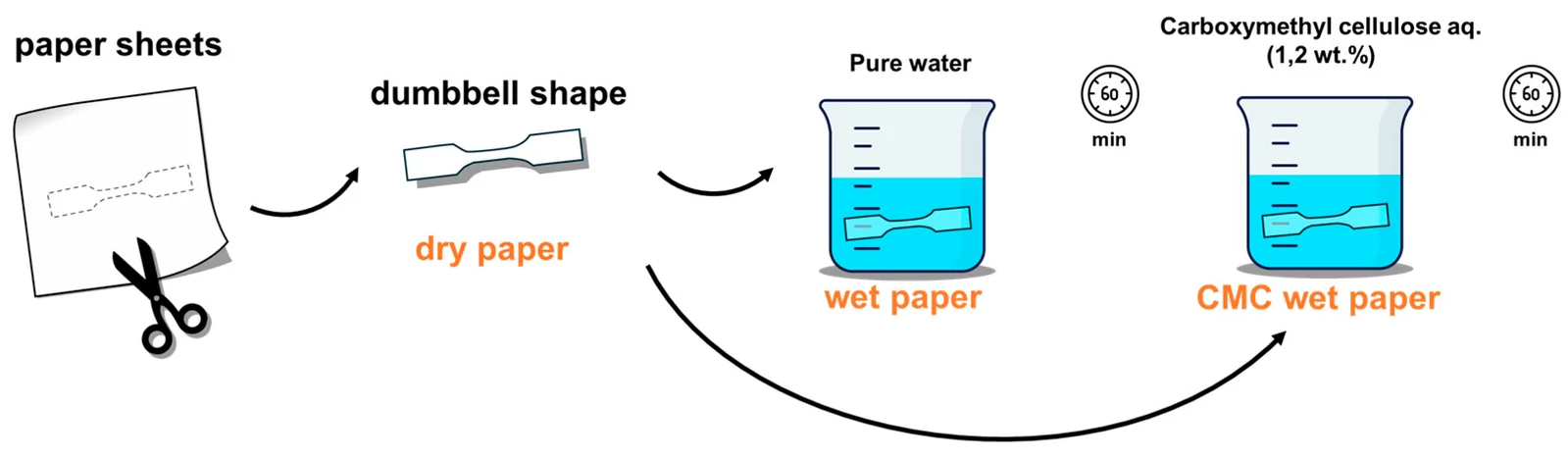
Schematic illustrations representing the samples used in this study: dry paper, wet paper, and CMC wet paper.

A commercially available paper (R100, Japan Pulp & Paper Co., Ltd., Chuo-ku, Japan) with an apparent density of 0.78 g/cm^3^ and a tensile index of 31.9 Nm/g was used for the mechanical tests in this study. Generally, there are two characteristic directions in paper sheets: the machine direction, in which the paper fibrils are oriented, and the cross direction, which is perpendicular to the fiber orientation [12]. In this study, the mechanical properties in both directions were investigated. Figure 1 shows the schematic illustrations representing the sample preparation in this study. A dry paper sheet was cut into samples with a dumbbell shape, which were used as the “dry paper”. Samples with a dumbbell shape were immersed in pure water for 60 min at room temperature, which were used as the “wet paper”. Samples with a dumbbell shape were immersed in 1 and 2 wt.% aqueous solution of sodium carboxymethyl cellulose with a degree of polymerization of 500 (CMC, Fujifilm Wako Chemicals, Osaka, Japan) for 60 min at room temperature, which were used as the “CMC wet paper”. The pH value of 2 wt.% aqueous solution of sodium carboxymethyl cellulose was 6.9, which was same as that of pure water used in this study.

### 2.2. Measurements of Weight and Size Changes

The weight changes due to the absorption of pure water or aqueous CMC solution at each concentration were measured by weighing the mass using an electronic balance (A&D Co., Ltd., GH–200, Toshima-ku, Japan). The measurement was carried out on three different samples, and the average value and standard error are shown in Table 1. The volume changes due to the absorption were also calculated by measuring the length of the samples using a digital microscope (LINKMICRO, LM206-B-JP, Shenzhen, China), and an image analysis was performed.

### 2.3. Mechanical Measurements

The stress−strain curves for the samples were measured at room temperature using a uniaxial tensile apparatus with a 500 N load cell (EZ−Test EZ−SX, Shimadzu Co., Kyoto, Japan). Each sample had a dumbbell shape, with a size of 4 mm wide and 26 mm long, and was stretched with tensile speeds of 2 and 10 mm/min in both the machine direction (MD) and the cross direction (CD). The thickness of the dry samples was measured by an electronic micrometer (QEM133−25 Niigata Seiki Co., Sanjo, Japan). The stress was the apparent stress, which was calculated from *σ* = *F*/*S*, where *F* is the load and *S* is the area of the cross-section of a paper sheet without deformation. The Young’s modulus for the samples was determined from the initial slope of the strain–stress curves. The strain energy density *U* was calculated by integrating at whole strains, i.e., at the region from natural length to a strain showing zero stress, and dividing by the volume of the sample. In this study, the strain energy density was analyzed separately before and after the peak of the stress–strain curve, i.e.,

(1) $$U=U_{1}+U_{2}$$

Here, *U*_1_ and *U*_2_ represent the strain energy density before and after the peak, respectively. These mechanical parameters were evaluated from measurements of three different samples, and their average value and standard errors are shown in the graph.

## 3. Results and Discussion

### 3.1. Weight and Size Changes

Table 1 shows the weight change, Δ*W*, and size changes, *L*_wet_/*L*_dry_ and *B*_wet_/*B*_dry_, of samples along the machine direction MD and CD due to the absorption of pure water or aqueous CMC solution at each concentration. The Δ*W* is calculated by the following equation:

(2) $$∆W=W_{\mathrm{wet}}+W_{\mathrm{dry}}$$

Here, *W*_wet_ and *W*_dry_ represent the weight of paper after and before immersion, respectively. The mean values of the Δ*W* in the MD and CD for wet paper were 0.0765 and 0.0650 g, respectively, which correspond to absorption ratios Δ*W*/*W*_dry_ of 2.3 and 2.1, respectively. The values of Δ*W*/*W*_dry_ for the CMC wet paper were high compared to those for pure water. Hypothesizing that the volume of the vacancy in the paper was not varied, due to the absorption and the density of CMC being 1.3 g/cm^3^, the difference in the Δ*W* between the paper in pure water and in 2 wt.% CMC was approximately 0.01 g for both the MD and CD, which cannot be explained by the difference in the density of CMC solution (~0.3 g/cm^3^). This indicates that the volume of the paper increased by absorbing these liquids [13]. For the samples in pure water, in the MD, the change in length parallel to the fiber orientation was 0.99 and that perpendicular to the fiber orientation was 1.04. This indicates that the water molecules penetrated between the fibers by being absorbed, expanding the space between the fibers. This fact was also confirmed by the data for the CD; the change in length parallel to the fiber orientation was 1.02 and that perpendicular to the fiber orientation was 1.04. The values of Δ*W*/*W*_dry_ for the wet CMC paper were higher than those in pure water, clearly exhibiting that a larger amount of solution than pure water was absorbed into the paper. An anisotropic expansion similar to that found in pure water was also observed in the CMC; that is, clear changes in the *L*_wet_/*L*_dry_ and *B*_wet_/*B*_dry_ were observed in the CD and MD, respectively, meaning that expansion only occurred perpendicular to the fiber orientation. Accordingly, it is considered that not only water molecules but also hydrated CMC chains penetrated between the fibers, expanding the space between the fibers. It should be mentioned that the size changes in the CMC solutions were almost the same as those in pure water, although the weight changes in CMC were far larger than those in pure water. As mentioned above, this result cannot be explained by the density of CMC, thus it may be due to the excess absorption of CMC into the paper. It is considered that this large expansion induced by CMC molecules is due to the osmotic pressure of CMC, a polyelectrolyte.

### 3.2. Mechanical Properties Along Machine Direction (MD)

**Figure 2:**
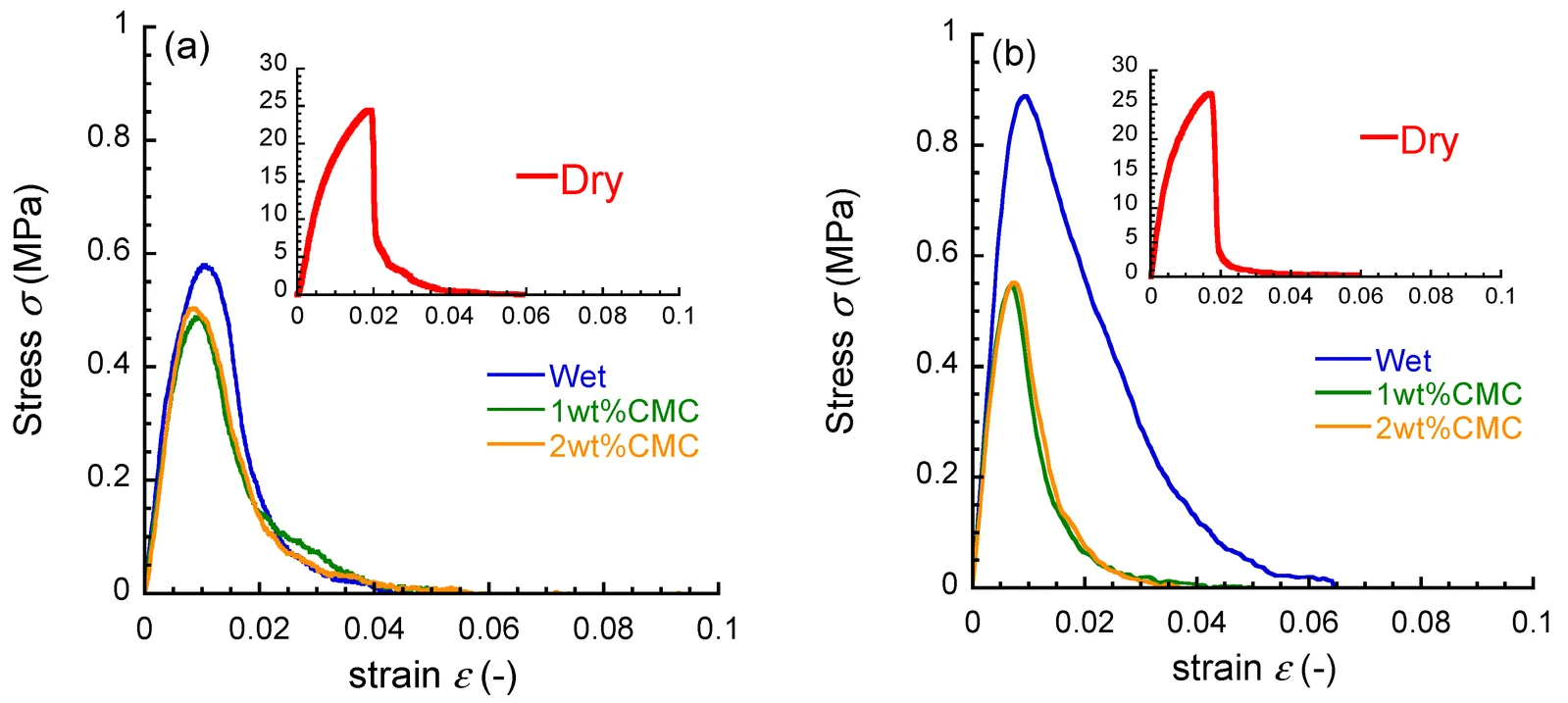
Stress–strain curves for wet paper and CMC wet papers at each concentration stretched along machine direction (MD) at tensile speeds of (**a**) 2 and (**b**) 10 mm/min. Inset: Dry paper.

Figure 2a shows the stress–strain curves for dry paper, wet paper, and CMC wet papers at each concentration at a tensile speed of 2 mm/min. The stress of dry paper increased proportional to the strain at *ε* < 0.008, showing elastic deformation, then showed a peak at *ε* ~ 0.017. At 0.008 < *ε* < 0.017, the stress exhibited slightly low values, showing plastic deformation. The magnitude of this plastic deformation altered depending on the absorption ratio of water in the paper [14]. In the linear viscoelastic region, no stress–strain hysteresis was observed for the dry paper. At *ε* > 0.017, the stress decreased rapidly, nearly equal to zero; however, it was not zero, meaning that the sample was not split into two objects. On the curve for the dry paper after the peak, many large spikes can be observed. This was caused by the breaking of the paper fibrils bridging over the cracks that hadh not broken at the peak. The stress for the wet paper and CMC wet papers increased linearly with the strain, i.e., elastic deformation at *ε* < 0.005 and *ε* < 0.005, respectively. Then, the wet paper and CMC wet papers showed broad peaks at *ε* ~ 0.009 and 0.008, respectively, after showing plastic deformation. Similar to the dry paper, there was no stress–strain hysteresis for the wet paper or CMC wet papers in the linear viscoelastic region. The stress for the wet paper decreased very gradually and even more gently at *ε* ~ 0.035; meanwhile, for the CMC wet papers it decreased gradually and even more gently at *ε* ~ 0.033. Similar to the dry paper, many spikes were observed, although they were very small. When the stress became zero, the strain was in the range of 0.040~0.060 for all samples. The regions of plastic deformation for the wet paper and CMC wet papers were 0.005 < *ε* < 0.009 and 0.005 < *ε* < 0.008, respectively.

Figure 2b shows the stress–strain curves for the dry paper, wet paper, and CMC wet papers at each concentration at a tensile speed of 10 mm/min. The shapes of the stress–strain curves for all samples are basically the same as those measured at 2 mm/min. A different feature can be observed at strains above the peak, where the stress becomes zero immediately, compared to that at 2 mm/min.

**Figure 3:**
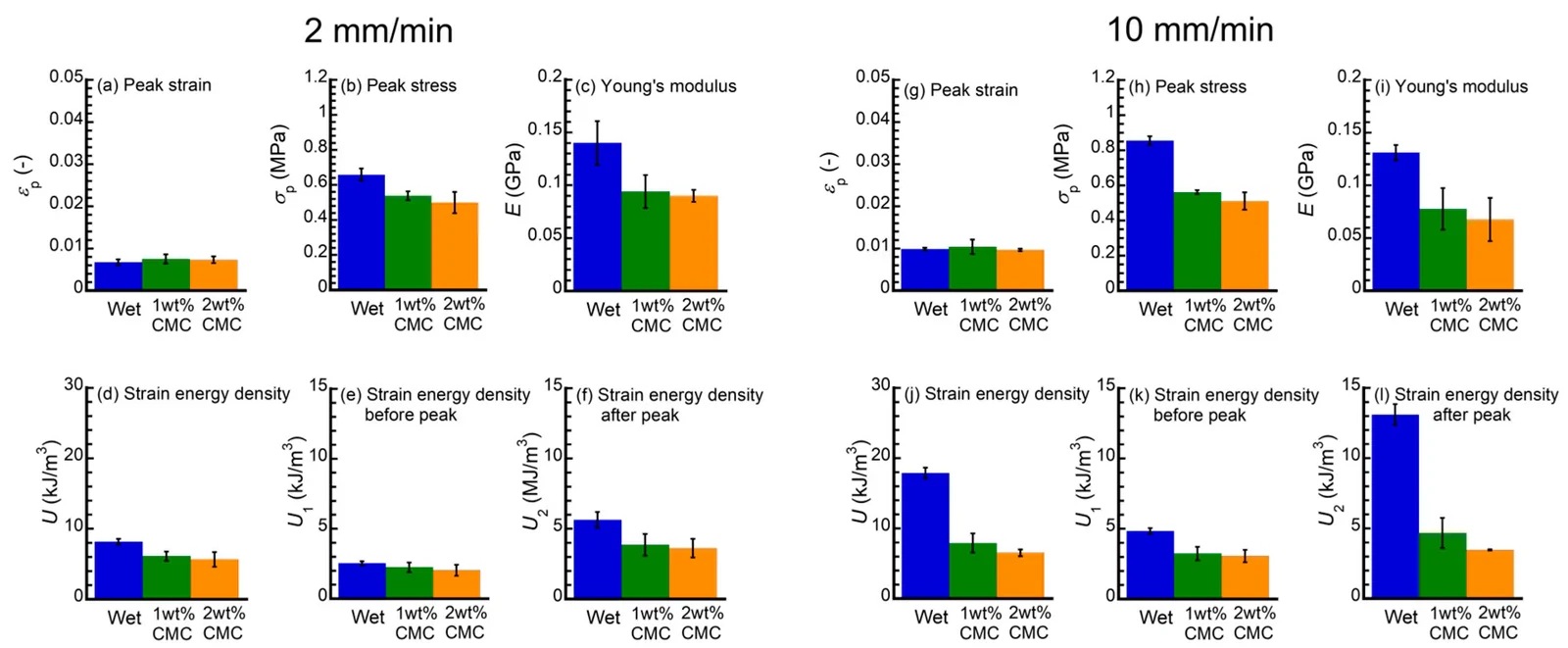
(**a**,**g**) Peak strain, (**b**,**h**) peak stress, (**c**,**i**) Young’s modulus, (**d**,**j**) total strain energy density, (**e**,**k**) strain energy density before peak, and (**f**,**l**) strain energy density after peak in MD for wet paper and CMC wet papers at each concentration, measured at tensile speeds of 2 (**left 6 panels**) and 10 mm/min (**right 6 panels**).

Figure 3a–f show the mechanical properties for the wet paper and CMC wet papers at each concentration measured at a tensile speed of 2 mm/min. For the dry paper at 2 mm/min, the peak strain *ε*_p_ and the peak stress *σ*_p_ were 0.018 ± 0.001 and 24.7 ± 0.7 MPa, respectively. The Young’s modulus *E* was 2.8 ± 0.1 GPa. The total strain energy density *U* was 362 ± 4 kJ/m^3^, and the strain energy density before the peak *U*_1_ was 266 ± 13 kJ/m^3^, corresponding to a value of *U*_1_/*U* of 74%.

The *ε*_p_ for the wet paper and CMC wet papers were almost the same and exhibited values approximately half of those of the dry paper (Figure 3a), which was due to the slippage between fibrils causing by wetting. Naturally, the *σ*_p_ for these papers was extremely low compared to that of dry paper (Figure 3b). The *σ*_p_ for the CMC wet papers was lower than that of wet paper, and it was almost the same regardless of the concentration, indicating that the fluid lubrication effect of CMC contributed to reducing the peak stress [15]. The *E* for the wet paper was 0.14 ± 0.02 GPa, which was 1/20 that of the dry paper. The Young’s modulus of paper is proportional to the volume fraction of paper fibers [16], meaning that it is also proportional to the number density of entanglement points of the fibers. Therefore, the reduction in *E* means that the number density of entanglement points for the wet paper was significantly reduced by absorbing water. It is considered that the disentanglement, by slipping out the fibers from the loop of entanglement point, was caused by the expansion due to the water absorption. The *U* for the wet paper, 1 wt.% CMC wet paper, and 2 wt.% CMC wet paper was 8.2, 6.1, and 5.7 kJ/m^3^, respectively (Figure 3d). The *U*_1_ for the wet paper, 1 wt.% CMC wet paper, and 2 wt.% CMC wet paper was 2.5, 2.2, and 2.0 kJ/m^3^, respectively, indicating that the CMC contributed to reduce the energy needed for disentanglement of fibers. The value of *U*_1_/*U* for the wet paper, 1 wt.% CMC wet paper, and 2 wt.% CMC wet paper was 31%, 38%, and 36%, respectively. Accordingly, approximately 40% of the *U* was mainly devoted to the elastic energy and friction energy of fibers, and the remaining 60% was mainly devoted to the friction energy between the fibers.

Figure 3g–l show the mechanical properties of wet paper and CMC wet papers at each concentration measured at a tensile speed of 10 mm/min. For the dry paper at 10 mm/min, the *ε*_p_ and the *σ*_p_ were 0.016 ± 0.0005 and 25.5 ± 0.7 MPa, respectively, which are almost the same as those measured at 2 mm/min. The *E* was 3.2 ± 0.2 GPa and the *U* was 355 ± 15 kJ/m^3^, which are nearly equal to those at 2 mm/min. The *U*_1_ was 259 ± 15 kJ/m^3^ and the value of *U*_1_/*U* was 73%, which are nearly the same as those at 2 mm/min. The slippage between paper fibers was not allowed in the dry paper, and the entanglement between the paper fibers was maintained because of high tensile speeds.

At 10 mm/min, there was no clear difference in the *ε*_p_ among these samples, while a clear difference in the *σ*_p_ was found between the wet paper and CMC wet papers, which is similar to the result at 2 mm/min. The *U* for the wet paper was 17.9 kJ/m^3^, which is twice that at 2 mm/min (=8.17 kJ/m^3^). These results suggest that there was not enough time for slipping out the fibers from the entanglement points at this tensile speed. Contrary to this, the *E* at 10 mm/min was almost the same as that at 2 mm/min for all samples. The values of *U* for the 1 wt.% and 2 wt.% CMC wet papers were 7.93 and 6.54 kJ/m^3^, respectively. The values of *U*_1_ for the wet paper and 1 wt.% and 2 wt.% CMC wet papers were 4.84, 3.24, and 3.07 kJ/m^3^, respectively, which correspond to values of *U*_1_/*U* of 27%, 42%, and 46%, respectively. These results reveal that the fluid lubrication effect by CMC is effective, particularly when further generation of entanglement points does not occur after the peak, and is independent of the CMC concentration.

**Figure 4:**
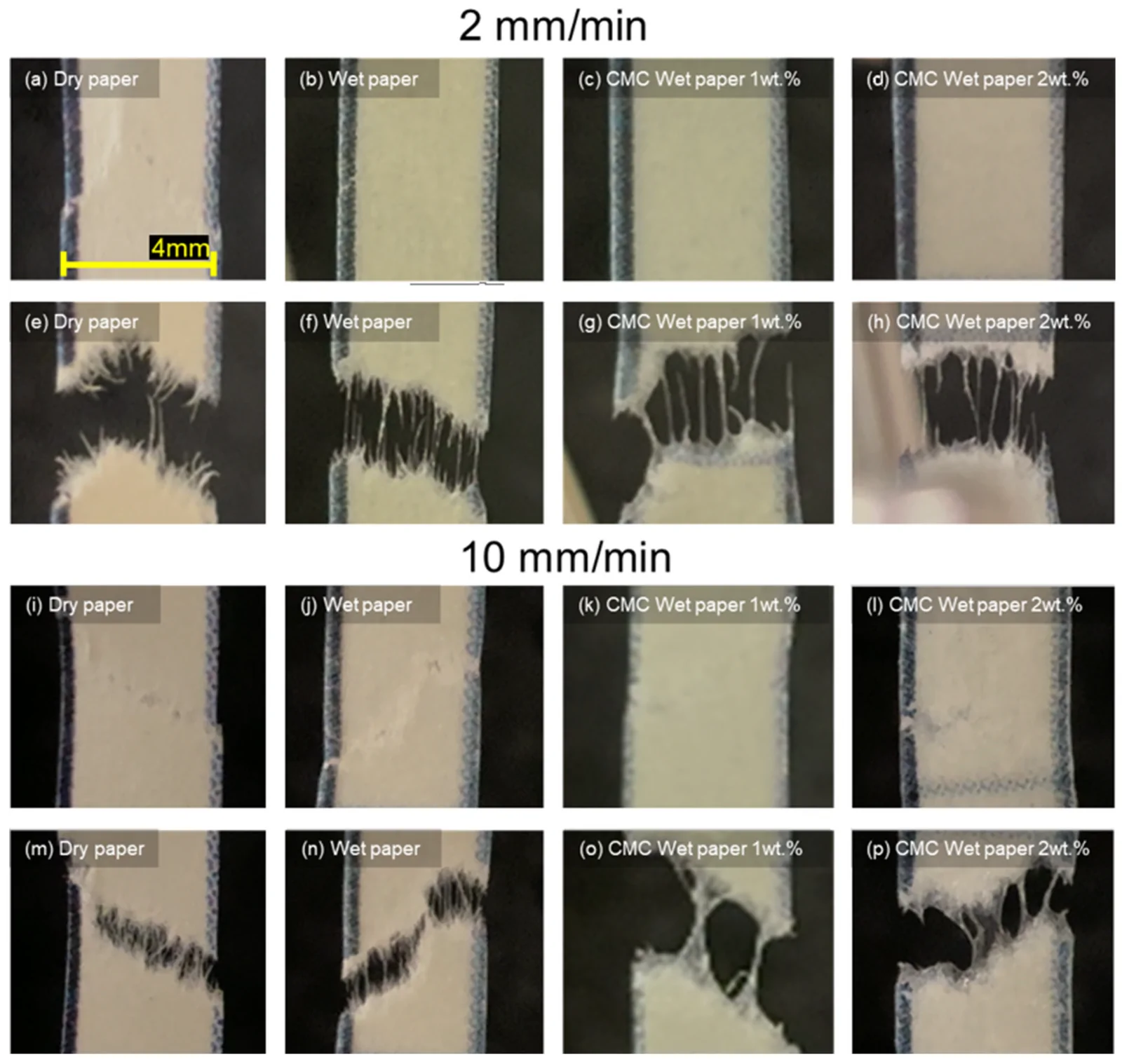
Photographs of the rupture behavior for dry paper, wet paper, and CMC wet papers at each concentration at ((**a**–**d**) and (**e**–**h**)) the peak strain and ((**i**–**l**) and (**m**–**p**)) after the peak strain at tensile speeds of 2 (**top 8 panels**) and 10 mm/min (**bottom 8 panels**).

Figure 4a–d show the photographs for the dry paper, wet paper, and CMC wet papers at the peak strain at a tensile speed of 2 mm/min. Only the dry paper showed a crack at the peak strain; however, no clear crack was observed for the other samples, even though the stress had already started to decrease. After the peak strain, all the samples had a crack and several fibrils connecting the upper and lower parts. Interestingly, it was observed that the number of paper fibers connecting the upper and lower parts was highest for the wet paper and it was nearly independent of the CMC concentration for the wet CMC papers.

Figure 4i–p show the photographs for the dry paper, wet paper, and CMC wet papers at the peak strain at a tensile speed of 10 mm/min. All the samples had a crack at the peak strain at this tensile speed. In all samples it was clearly observed that at the cracked area the length and the number of fibers pulled out from the inside of the paper was short and less when compared to those at 2 mm/min. This means that many entanglement points were generated and functioned, and the number of broken fibers were more than those of slipped between fibers at 10 mm/min. This would be a reason why the total energy at 2 mm/min in Figure 3 is lower than that at 10 mm/min for all samples. It is valuable to note that, for the CMC wet papers, the fibers pulled out of the rupture surface formed bundles covered by CMC solution, suggesting that a strong interaction occurred between the paper fibers and CMC chains, resulting in a significant contribution to the fluid lubrication.

**Figure 5:**
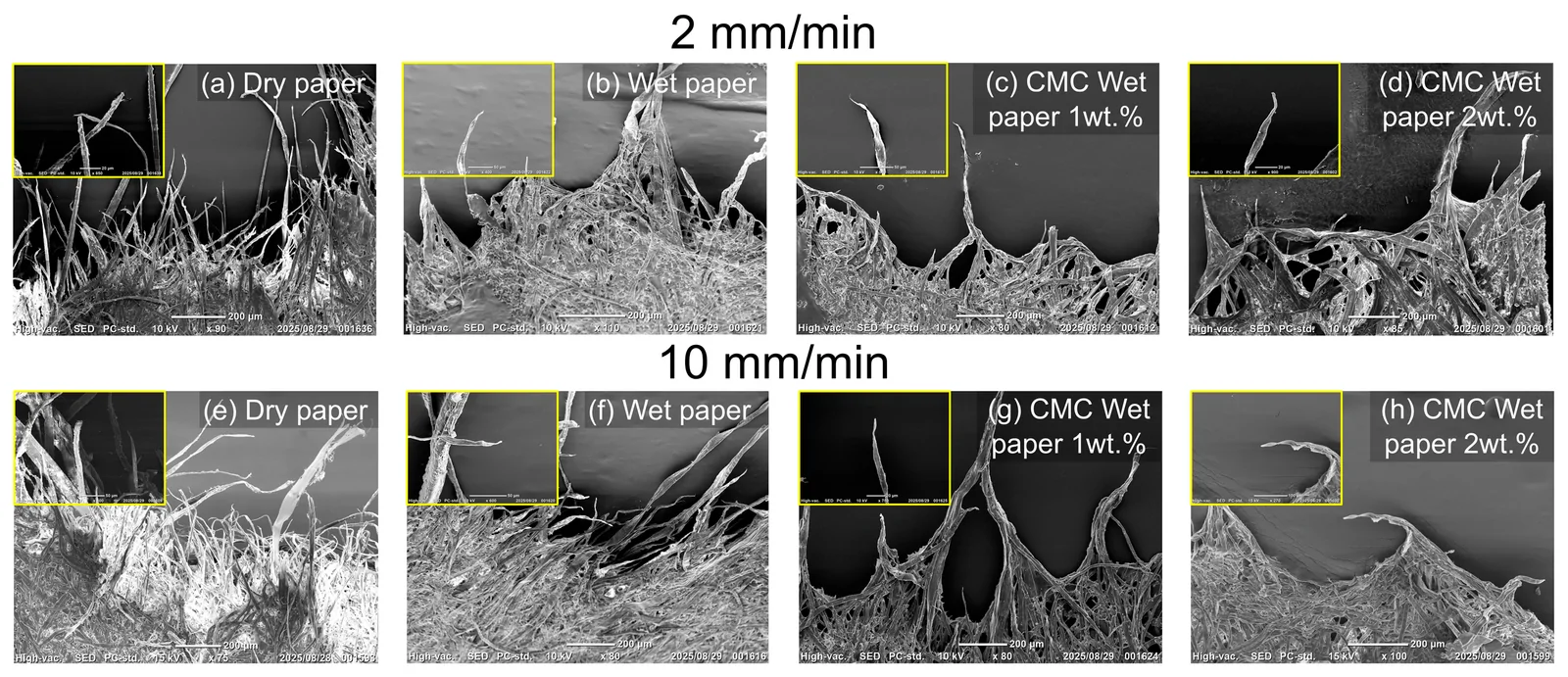
SEM photographs of rupture surface for (**a**,**e**) dry paper, (**b**,**f**) wet paper, and (**c**,**d**,**g**,**h**) CMC wet papers at each concentration at tensile speeds of 2 (**top 4 panels**) and 10 mm/min (**bottom 4 panels**).

Figure 5 shows the SEM photographs for the dry paper, wet paper, and CMC wet papers at tensile speeds of 2 and 10 mm/min. A clear difference between the fiber ends of dry and wet papers is observed in the inset photos: the shape of the fiber end for the dry paper is thick, while for both the wet and wet CMC they are thin and sharp. This strongly indicates that the dry fibers mainly broke within the fibers with less slippage between fibers, and the wet fibers mainly broke by slippage between the fibers while maintaining their fiber shape, which supports the information obtained from the stress–strain curves. It was also observed at the rupture surface that most of fibers were single fibers in the dry paper and were in bundles in the wet papers, which agrees with the macroscopic morphology from the photographs of the crack in Figure 4. Similar characteristics were observed at 10 mm/min for both the shape of the fiber ends and the number of fibers.

### 3.3. Mechanical Properties Along Cross Direction (CD)

**Figure 6:**
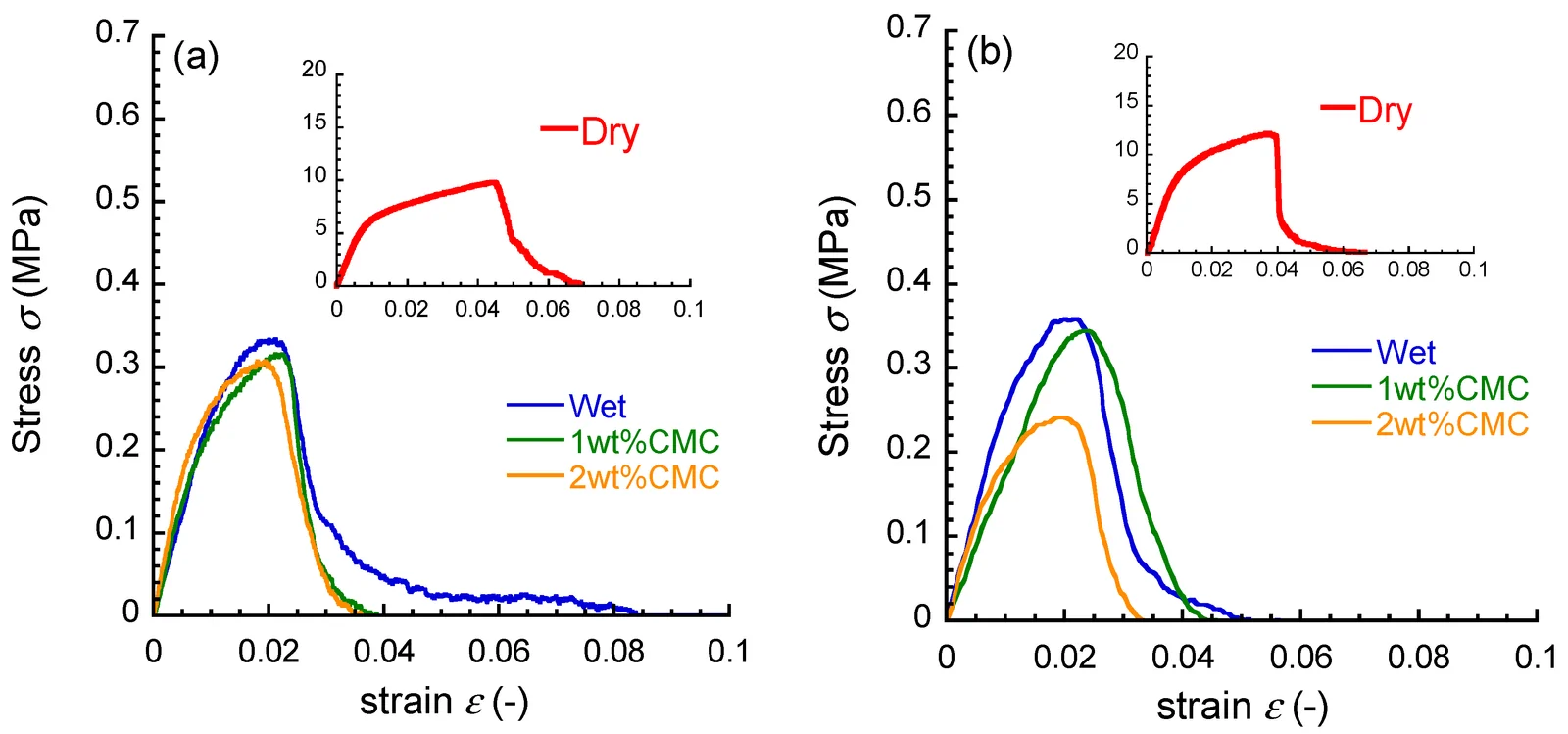
Stress–strain curves for wet paper and CMC wet papers at each concentration stretched along cross direction (CD) at tensile speeds of (**a**) 2 and (**b**) 10 mm/min. Inset: Dry paper.

Figure 6a exhibits the stress–strain curves for the dry paper, wet paper, and CMC wet papers at each concentration at a tensile speed of 2 mm/min. The stress for the dry paper increased proportional to the strain at *ε* < 0.005, showing elastic deformation, then showed a peak at *ε* ~ 0.045. At 0.005 < *ε* < 0.045, the stress exhibited significantly low values deviating from the linear, showing plastic deformation. The plastic region was very large compared to that in the MD, which was due to the macroscopic deformation of fibers. The plastic deformation in the MD was not significant since the mechanical response of paper sheets along the MD was mainly due to stress generation by the deformation of individual fibers [17]. At *ε* > 0.045, the stress decreased rapidly; however, it was not zero, having the same behavior as in the MD. At strains higher than the peak, many spikes were observed, although they were relatively small compared to those in the MD. In the linear viscoelastic regime, where the stress was proportional to the strain, no mechanical hysteresis was found in the curve, indicating that the paper deformed without slippage between the fibers [18]. In the plastic region, the stress was under-deviated from the linear, which was evidenced by the fact that the entanglement point disappeared due to the slipping fibers. However, the stress continued to increase with an increasing strain in this region [19]. Therefore, this indicates that new entanglement points were generated although the chain slipped out from the entanglement point. At strains above the peak, the breaking and/or slipping of fibers dominated the mechanical properties of the papers [20]. The stress for the wet paper and CMC wet papers increased linearly with the strain, i.e., elastic deformation at *ε* < 0.006 and *ε* < ~0.005, respectively. Then, the wet paper and CMC wet papers showed broad peaks immediately after the region of elastic deformation. After the peak, the stress for the wet paper decreased significantly and decreased gradually at *ε* ~ 0.03, and even more gently at *ε* ~ 0.05. The stress for the CMC wet papers decreased significantly and decreased gently at *ε* ~ 0.03. Similar to the dry paper, many spikes were observed for both the wet paper and CMC wet papers, although they were very small. The strain at which the stress dropped to zero was around 0.08 for the wet paper and the values of the strain ranged from 0.03 to 0.08. The regions of plastic deformation for the dry paper, wet paper, and CMC wet papers were 0.005 < *ε* < 0.045, 0.006 < *ε* < 0.022, and 0.005 < *ε* < 0.022, respectively.

Figure 6b shows the stress–strain curves for the dry paper, wet paper, and CMC wet papers at each concentration at a tensile speed of 10 mm/min. For the dry paper, the curve at 10 mm/min exhibits a narrow plastic deformation region and an instantaneous decrease in the stress after the peak compared to at 2 mm/min. The narrowing of the plastic deformation region was probably due to the fact that the number density of entanglement points generated by fiber slippage is reduced under high tensile speed; this is cause, for example, by breaking fibers without slippage. For the other samples, the shapes of the stress–strain curves at 10 mm/min are basically the same as those at 2 mm/min.

**Figure 7:**
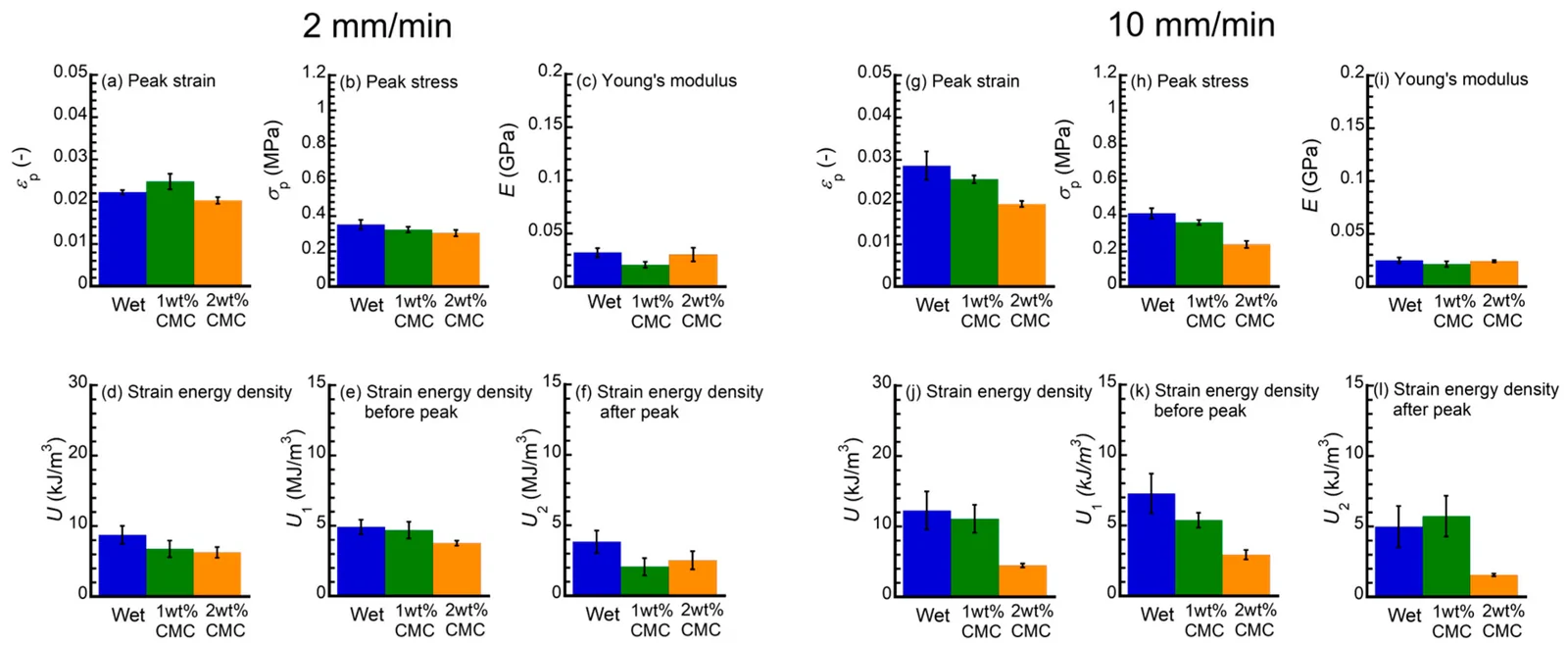
(**a**,**g**) Peak strain, (**b**,**h**) peak stress, (**c**,**i**) Young’s modulus, (**d**,**j**) total strain energy density, (**e**,**k**) strain energy density before peak, and (**f**,**l**) strain energy density after peak in CD for wet paper and CMC wet papers at each concentration stretched at tensile speeds of 2 (**top 6 panels**) and 10 mm/min (**bottom 6 panels**).

Figure 7a–f show the mechanical properties for the wet paper and CMC wet papers at each concentration measured at a tensile speed of 2 mm/min. For the dry paper at 2 mm/min, the peak strain *ε*_p_ and the peak stress *σ*_p_ was 0.047 ± 0.001 (~2.6 times of MD) and 9.06 ± 0.4 MPa (~1/3 of MD), respectively. The Young’s modulus *E* was 0.89 ± 0.08 GPa (~1/3 of MD), suggesting fewer entanglement points than in the MD. Thus, although the paper stretched in the CD direction, it exhibited low stress. The total strain energy density *U* was 375 ± 30 kJ/m^3^, which is nearly the same as that in the MD (=362 ± 5 kJ/m^3^). The strain energy density before the peak *U*_1_ was 314 ± 10 kJ/m^3^, corresponding to a value of *U*_1_/*U* of 85%, i.e., 10% higher than in the MD.

The *ε*_p_ of the wet paper was approximately half of that of dry paper, similar to the result in the MD. The *σ*_p_ decreased gradually with an increasing CMC concentration. The *E* for the wet paper was 0.032 ± 0.040 GPa, which was 1/28 that of the dry paper. This value was ~1/3 of that in the MD, probably due to the anisotropic expansion, as discussed in Table 1. The values of *E* for the CMC wet papers were almost equal to that of wet paper, suggesting that the number density of entanglement points in the CMC wet papers was nearly equal to that of wet paper; actually, isotropic expansion was observed in CD. The values of *U* for the wet paper, 1 wt.% CMC wet paper, and 2 wt.% CMC wet paper were 8.7586, 6.7583, and 6.2882 kJ/m^3^, respectively, which were similar values to those in the MD. The values of *U*_1_ for the wet paper, 1 wt.% CMC wet paper, and 2 wt.% CMC wet paper were 4.95, 4.69, and 3.76 kJ/m^3^, respectively, which were slightly higher than those in the MD.

Figure 7g–l show the mechanical properties of wet paper and CMC wet papers at each concentration measured at a tensile speed of 10 mm/min. For the dry paper at 10 mm/min, the peak strain *ε*_p_ and the peak stress *σ*_p_ was 0.043 ± 0.003 (~0.9 times of 2 mm/min) and 12.6 ± 0.5 MPa (1.4 times of 2 mm/min), respectively. The *E* was 1.0 ± 0.1 GPa (1.1 times of 2 mm/min) and the *U* was 439 ± 40 kJ/m^3^ (1.2 times of 2 mm/min). The origin of the observed high energy was a matter of the time required for disentanglement of fibers, as mentioned above. The *U*_1_ was 373 ± 32 kJ/m^3^ (1.2 times of 2 mm/min), which corresponds to a value of 90% (73% at 2 mm/min).

The *ε*_p_ and *σ*_p_ for the CMC wet papers clearly decreased with the CMC concentration, and this behavior was not seen at 2 mm/min. The *E* was almost the same for the wet paper and CMC wet papers. The values of *U* for the wet paper, 1 wt.% CMC wet paper, and 2 wt.% CMC wet paper were 12.3, 11.1, and 4.48 kJ/m^3^, respectively, which were higher than those at 2 mm/min. The values of *U*_1_ for the wet paper, 1 wt.% CMC wet paper, and 2 wt.% CMC wet paper were 7.28, 5.39, and 2.93 kJ/m^3^, respectively, which were slightly higher than those at 2 mm/min.

Comparing the CD with MD at 10 mm/min, the *ε*_p_ values in the CD were high and the values of *σ*_p_ were low compared to those in the MD, which was due to the macroscopic deformation mentioned above. It should be noted that the values of *E* were also low compared to those in the MD. This means that the number density of entanglement points in the CD was fewer even at strains of linear viscoelasticity, where the slippage of fibers did not occur.

**Figure 8:**
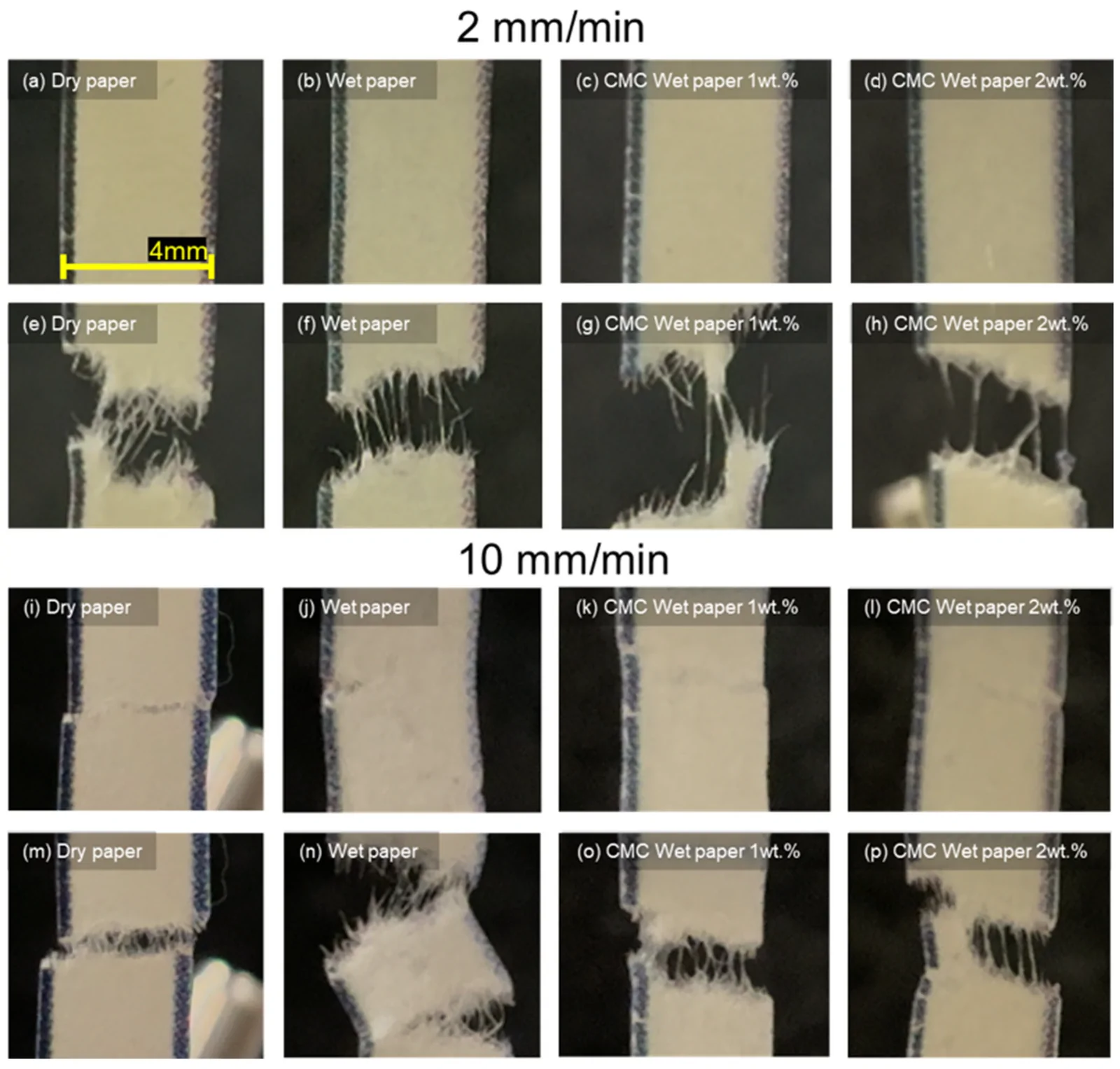
Photographs of rupture of dry paper, wet paper, and CMC wet papers at each concentration at ((**a**–**d**) and (**e**–**h**)) peak strain and ((**i**–**l**) and (**m**–**p**)) after the peak strain when stretched at tensile speeds of 2 (**top 8 panels**) and 10 mm/min (**bottom 8 panels**).

Figure 8a–p show the photographs of dry paper, wet paper, and CMC wet papers at tensile speeds of 2 and 10 mm/min. Different from the rapture behavior in the MD, no cracks were observed in any sample at the peak strain at 2 mm/min, which is attributed to the sufficient slipping of fibers without breaking in wide region of plastic deformation. After the peak strain, many fibers were observed at the rapture surface. At 10 mm/min, all the samples exhibited a crack at the peak strain, and multiple fibers connecting the upper and lower parts were observed in the cracked area.

**Figure 9:**
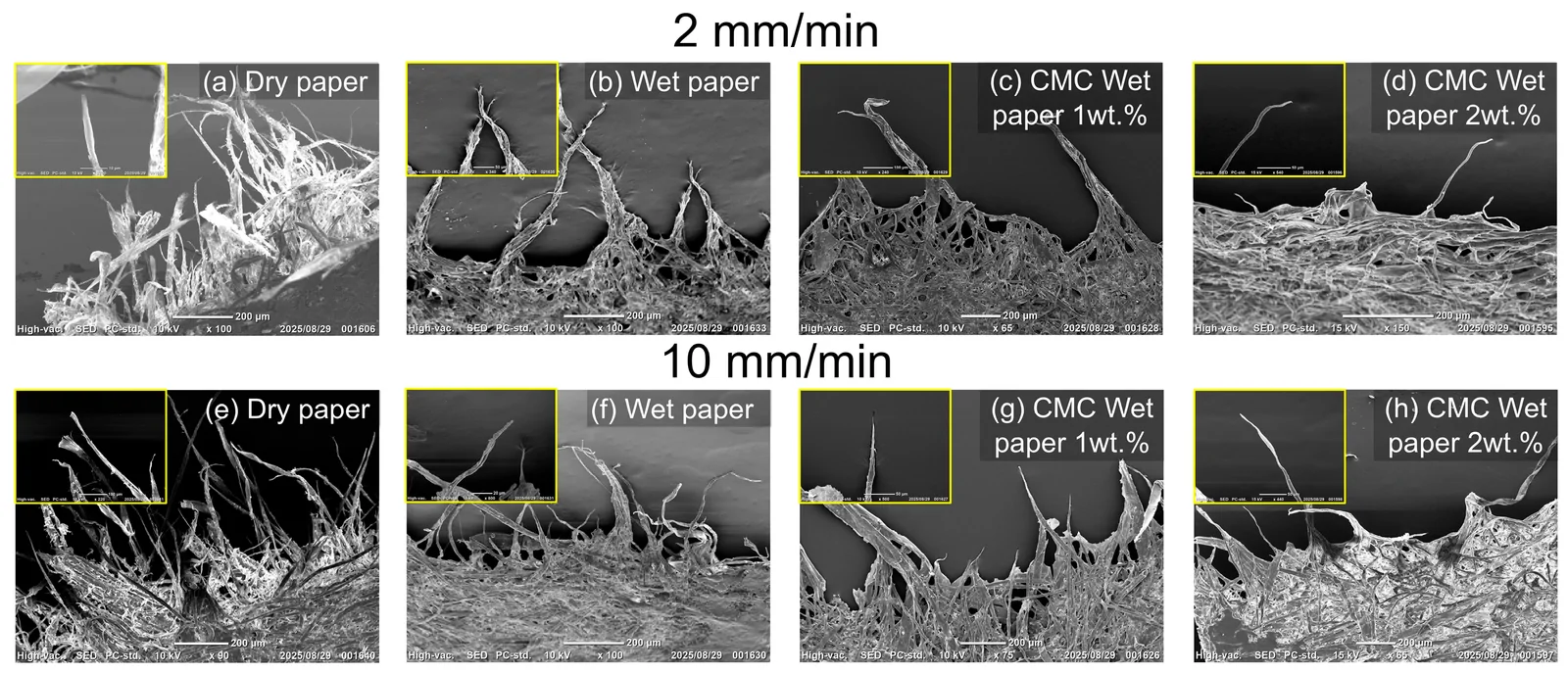
SEM photographs of rupture surface for (**a**,**e**) dry paper, (**b**,**f**) wet paper, and (**c**,**d**,**g**,**h**) CMC wet papers at each concentration at tensile speeds of 2 (**top 4 panels**) and 10 mm/min (**bottom 4 panels**).

Figure 9 shows the SEM photographs for the dry paper, wet paper, and CMC wet papers at tensile speeds of 2 and 10 mm/min. The difference seen in the fiber ends between the dry paper and wet papers was also observed in the CD. An observed feature of fibers at the rupture surface was that the most of fibers were single fibers in the dry paper and were in bundles in the wet papers. Many more fibers were seen in the CD compared to the MD; this is reasonable because the breaking happened within fibers, and a single fiber produced two fiber ends upon breaking, thereby increasing the number of fiber ends. Similar features in the shape of the fiber end were seen at a tensile speed of 10 mm/min.

**Figure 10:**
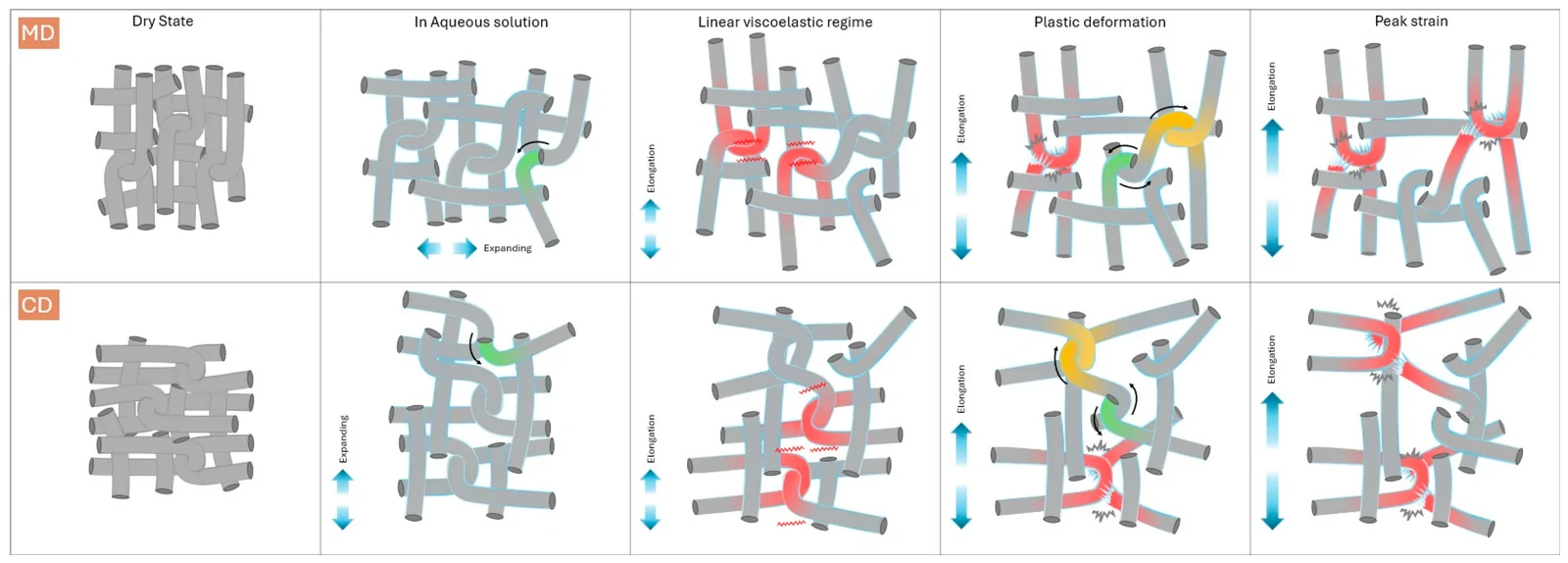
Schematic illustration representing the status of paper chains in various conditions for dry paper, wet paper, and CMC wet papers in machine direction (**tops**) and cross direction (**bottoms**). The moving direction of fibers is indicated by arrows.

Figure 10 shows a schematic illustration representing the rupture behavior of dry paper, wet paper, and CMC wet papers stretched along the MD and CD. In water and the aqueous CMC solution, the papers elongate along the direction perpendicular to the fiber orientation, i.e., horizontal for the MD and vertical for the CD, as shown in the figure. While this is occurring, some fibers slip out from the entanglement points (green part). For the papers immersed in CMC, this effect is significant due to the large expansion originating from the osmotic pressure. In the linear viscoelastic regime, an applied force acts on the entanglement points without showing slippage (red part). In the region of plastic deformation, there are fibers that break or slip at the entanglement point and the slipped fibers make new entanglement points (yellow part). The fluid lubrication effect by the CMC molecules is significant in this region and it contributes to the low friction between paper fibers. At the peak strain, only breaking and slippage occurs and no entanglement points are generated, which contribute to further increasing the apparent stress.

## 4. Conclusions

The effect of carboxymethyl cellulose on the mechanical properties and the rupture behavior of paper was investigated through the immersion-induced size and gravimetric changes and uniaxial tensile tests. It was found that the paper sheets expanded mainly in the direction perpendicular to the fiber orientation by water absorption, and the expansion was significant for the papers immersed in CMC solution. This led to a reduction in the number density of originally existing entanglement points, resulting in a decrease in the mechanical properties, such as Young’s modulus. The significant disentanglement in the CMC was caused by the large expansion, which was due to the osmotic pressure of counterions of CMC adhered onto the paper fibers. The strain energy density demonstrated that the fluid lubrication effects of water and CMC aqueous solutions significantly reduced the friction between fibers. Accordingly, wetting the paper reduced the number of fibers that broke. Based on the above experimental findings, it is found that immersing paper in a CMC aqueous solution is an effective way to reduce or control the interactions between paper fibers, and this could have great potential for applications in conservation science. Furthermore, all samples exhibited low elongation and high stress in the MD direction, but high elongation and low stress in the CD direction. Therefore, peeling in the MD direction allows for the application of strong force at the peeling interface. Although its practical utility remains unclear at the moment, this finding could also be important in conservation science and restoration of cultural assets and paintings.

## Figures and Tables

**Table 1:** (a) Weight and (b) size changes in samples along machine direction (MD) and cross direction (CD) due to absorption of pure water or aqueous CMC solution at each concentration.

(a)				*W*_dry_ (g)	*W*_wet_ (g)	ΔW (g)		ΔW/*W*_dry_ (-)
		MD	Pure Water	0.0327	0.1090	0.0765 ± 0.0040		2.3 ± 0.1
			1 wt.% CMC	0.0319	0.1090	0.0772 ± 0.0010		2.4 ± 0.1
			2 wt.% CMC	0.0317	0.1170	0.0849 ± 0.0020		2.7 ± 0.1
		CD	Pure Water	0.0315	0.0964	0.0650 ± 0.0030		2.1 ± 0.1
			1 wt.% CMC	0.0316	0.1016	0.0700 ± 0.0050		2.2 ± 0.2
			2 wt.% CMC	0.0312	0.1080	0.0767 ± 0.0020		2.5 ± 0.1
	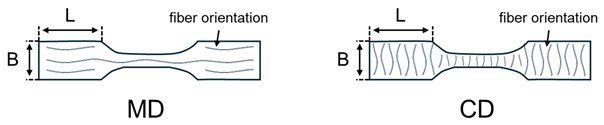							
(b)			*L*_dry_ (cm)	*L*_wet_ (cm)	*L*_wet_/*L*_dry_ (-)	*B*_dry_ (cm)	*B*_wet_ (cm)	*B*_wet_/*B*_dry_ (-)
	MD	Pure Water	1.64	1.62	0.99 ± 0.00	1.01	1.05	1.04 ± 0.00
		1 wt.% CMC	1.65	1.64	0.99 ± 0.00	1.01	1.06	1.05 ± 0.01
		2 wt.% CMC	1.65	1.62	0.98 ± 0.00	1.01	1.05	1.04 ± 0.01
			*L*_dry_ (cm)	*L*_wet_ (cm)	*L*_wet_/*L*_dry_ (-)	*B*_dry_ (cm)	*B*_wet_ (cm)	*B*_wet_/*B*_dry_ (-)
	CD	Pure Water	1.68	1.74	1.04 ± 0.00	0.97	0.98	1.02 ± 0.01
		1 wt.% CMC	1.67	1.74	1.04 ± 0.00	1.01	1.06	1.05 ± 0.01
		2 wt.% CMC	1.66	1.74	1.05 ± 0.01	0.98	0.98	0.99 ± 0.01

## Data Availability

The original contributions presented in this study are included in the article. Further inquiries can be directed to the corresponding author.
